# Vasovagal Syncope and Transient Asystole During Peripheral Nerve Blockade

**DOI:** 10.7759/cureus.39337

**Published:** 2023-05-22

**Authors:** Alexander M DeLeon, Joseph Rivera-Monfeli, Talal A Akbar, Bruno DeCaria

**Affiliations:** 1 Anesthesiology, Northwestern University Feinberg School of Medicine, Chicago, USA

**Keywords:** atropine, ephedrine, peripheral nerve blockade, vasovagal syncope, asystole

## Abstract

Vasovagal syncope has been associated with chronic pain procedures, phlebotomy, and musculoskeletal injections. While vasovagal syncope is commonly associated with interventional pain procedures, its occurrence during peripheral nerve block procedures has not been reported. We report a case of vasovagal syncope leading to transient asystole in a patient undergoing a lower extremity peripheral nerve block procedure. The episode resolved after halting the procedure and administering ephedrine, atropine, and intravenous fluids.

## Introduction

Vasovagal syncope is defined as a rapid drop in heart rate and blood pressure, often in response to a stimulating procedure. While vasovagal syncope is commonly associated with interventional pain procedures, its association with peripheral nerve blocks has not been documented [1].

We present a case of vasovagal syncope leading to transient asystole in a patient undergoing an ultrasound-guided adductor canal block. The episode occurred before the injection of local anesthetic for the nerve block, thus ruling out local anesthetic systemic toxicity as a cause. Our report represents the first case of vasovagal syncope leading to transient asystole in a patient undergoing peripheral nerve blockade. We will discuss the risk factors and treatment of vasovagal syncope.

## Case presentation

A 25-year-old male with a BMI of 32.11 kg/m^2^ presented for left foot tarsometatarsal arthrodesis. The surgery occurred at 3:30 p.m., and the patient's final clear liquid intake consisted of water three hours before surgery. His last solid intake was the previous night at 9:20 p.m. His past medical history was significant for a history of migraine headaches and mild asthma. His height was 5'10", and his weight was 101.5 kg. His physical exam was non-contributory. The anesthesia team planned to perform an ultrasound-guided adductor canal block followed by an ultrasound-guided sciatic nerve block. The nerve block was conducted in the preoperative holding area. The patient was monitored with ASA standard monitoring and received supplemental oxygen.

**Figure 1:**
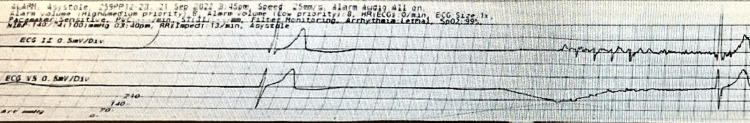
Rhythm strip Rhythm strip demonstrating eight to 10-second pauses between QRS complex and a reading of "asystole."

**Figure 2:**
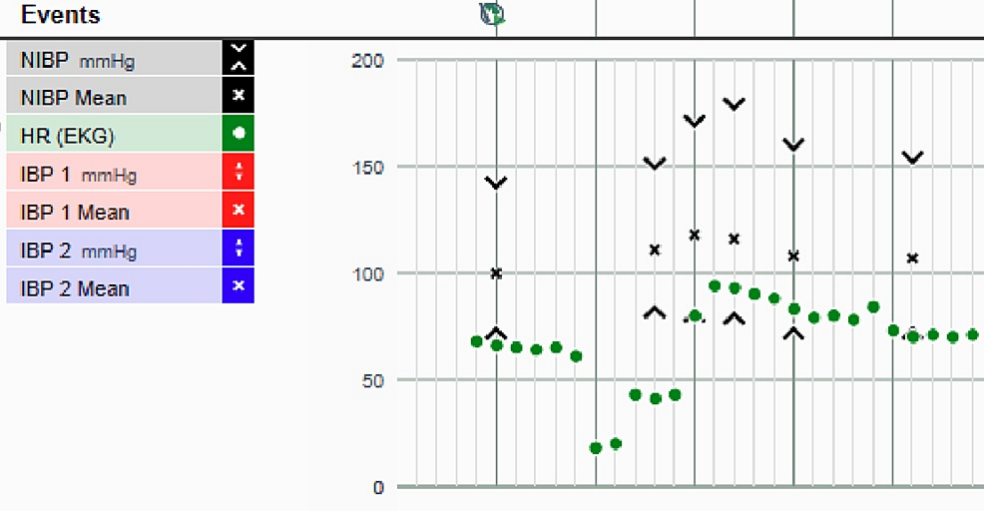
Anesthesia record Anesthesia record during the peripheral nerve block indicates an undetectable blood pressure and a drop in heart rate from 60 to 10 bpm. IBP: invasive blood pressure; NIBP: non-invasive blood pressure; HR: heart rate.

Premedication before the adductor canal block included 2 mg of midazolam and 50 mcg of fentanyl. Five minutes after initiating the nerve block, the patient complained of "not feeling well." The nerve block was halted immediately. The rhythm strip demonstrated eight to 10-second pauses between QRS complexes with the alarm for "asystole" being triggered (Figure 1). The patient was immediately placed in the Trendelenburg position, and his intravenous fluid rate was increased to free flow. The anesthesia record showed a heart rate as low as 10 bpm (Figure 2). The patient was unresponsive at this time to verbal and physical stimulation, and a "code blue" was initiated with the emergency crash cart immediately brought to the bedside.

Ephedrine 30 mg was administered intravenously, followed by 0.5 mg of atropine. Within three minutes of the onset of the episode, the heart rate recovered to 43 bpm with a blood pressure of 149/84 mmHg. The patient's symptoms resolved with increased heart rate and blood pressure. With the rapid resolution of the event, chest compressions were not needed.

After the patient's symptoms resolved and his hemodynamics were stable, the peripheral nerve blocks continued, and the patient underwent uneventful outpatient surgery. The recovery room course was per usual protocol with no additional monitoring, given that the preoperative event had resolved. The patient demonstrated no long-term sequelae at a three-month follow-up.

## Discussion

The pathophysiology of vasovagal syncope is poorly understood. It has been suggested that procedure-related vasovagal syncope is a cardioprotective reflex triggered by a hypercontractile heart in the setting of inadequate preload, like the Bezold-Jarisch reflex [1]. Vasovagal syncope has been reported in the anesthesia literature in association with chronic pain procedures and venous cannulation. The current literature lacks reports of vasovagal syncope associated with the performance of peripheral nerve block procedures.

Malave et al. [1] reviewed vasovagal reactions associated with interventional pain management procedures. Risk factors include previous history of vasovagal reactions, male gender, age < 65 years, baseline hypotension, dehydration, and baseline anxiety [2,3]. Preprocedural chronic pain scores were also a risk factor for developing vasovagal syncope. Kennedy et al. [4] demonstrated an increased incidence of pain procedure-related vasovagal syncope when preprocedural pain scores were lower than five out of 10 than when preprocedural pain scores were higher. Our reported patient was visibly anxious and likely dehydrated, related to his nothing by mouth (NPO) status.

The orthopedic surgery literature also contains reports of office-based procedures related to vasovagal syncope. Al-Assam et al. [5] reported the results of a study on 2,462 ultrasound-guided musculoskeletal injections performed by orthopedic surgeons. Their group observed a rate of syncope of 2.3%. The orthopedic study demonstrated that younger patients < 65 years old were more likely to experience vasovagal syncope, as was the case in chronic pain patients.

Vasovagal reactions are common with blood donation and venous cannulation. Pavlin et al. [6] reported an incidence of 10.6% among patients undergoing venous cannulation. The highest risks were younger patients less than 40 years of age and those with a previous history of fainting episodes.

Many prophylactic interventions have been reported for the prevention of vasovagal syncope. These include preprocedural sedation, anxiolysis, antimuscarinics, and intravenous hydration [1]. Sedation and anxiolytics are routinely used at our institution when placing peripheral nerve blocks. Prophylactic antimuscarinic medication use is more controversial and is of questionable utility [1].

The treatment of vasovagal syncope includes Trendelenburg position, ephedrine, glycopyrrolate, and atropine. The dosing and timing of each therapeutic agent depend on the severity and responsiveness to initial therapies. After the vasovagal syncope episode ended, our patient underwent both the nerve block and surgical procedure. The decision to proceed with the surgery was based on the rapid resolution of the event, the patient's hemodynamic stability, and the desire of the patient to continue with surgery.

This report's primary limitation is that case reports are based on a single patient, limiting the findings' generalizability. A larger sample size or additional case reports would strengthen the evidence.

## Conclusions

Vasovagal syncope may occur in patients undergoing peripheral nerve block procedures for ambulatory orthopedic surgery. The risk factors include male gender, age < 65 years, previous history of vasovagal syncope, low baseline blood pressure, high anxiety level, and low preprocedural pain score. Clinicians should be prepared to promptly treat vasovagal syncope by ensuring intravenous access and having medications like ephedrine and atropine readily available, as well as providing fluid administration. This approach can help prevent long-term complications.
